# Explainable temporal graph-based CNNs for predicting hip replacement risk using EHR data

**DOI:** 10.1136/bmjdhai-2025-000136

**Published:** 2026-01-27

**Authors:** Zoe Hancox, David Wong, Sarah R Kingsbury, Philip G Conaghan, Andrew Clegg, Samuel D Relton

**Affiliations:** 1 University of Leeds, Leeds, UK; 2 Leeds Institute of Health Sciences, University of Leeds, Leeds, UK; 3 NIHR Leeds Biomedical Research Centre, Leeds, UK; 4 Bradford Institute for Health Research, Bradford, UK

**Keywords:** Artificial intelligence, Decision Support Systems, Clinical, Electronic Health Records, Primary Health Care

## Abstract

**Objective:**

To develop and compare four explainable artificial intelligence methods to visualise the influence of electronic health records (EHRs) on predicting hip replacement risk.

**Methods and analysis:**

We used a pretrained temporal graph-based convolutional neural networks (TGCNN) model to generate explainable graph visualisations through four methods: the original gradient-weighted class activation mapping (Grad-CAM) applied to graphs, a modified Grad-CAM using absolute weights (Grad-CAM (abs)), sliding element-wise multiplication of feature maps with patient graph inputs (fm-act) and of 3D convolutional neural networks filters/kernels with patient graph inputs (edge-act). These methods visually explain the TGCNN model’s predictions regarding a person’s risk of needing a hip replacement within 5 years, based on clinical codes from EHRs. We evaluated these models through human qualitative analysis studies, sensitivity quantification, edge detection bias and sparsity.

**Results:**

The edge-act methods performed best in terms of graph sparsity and model sensitivity. Subgraph analysis indicated that prescriptions highly influenced predictions. Clinicians found the visualisations useful for explaining model predictions but too complex for clinical decision-making, particularly with extensive patient EHRs.

**Conclusions:**

The fm-act and Grad-CAM (abs) methods led to graphs with zero sparsity; these graphs could be difficult to interpret if the patient has a long EHR history. The edge-act median method had the highest sparsity; therefore, this method might be the easiest to interpret for long EHR histories. We improved the explainability of hip replacement risk predictions using four post hoc methods on the TGCNN model. Further refinement could enhance their utility in clinical decision-making.

**WHAT IS ALREADY KNOWN ON THIS TOPIC**

- Artificial intelligence (AI) is becoming more widely used in healthcare applications. Explainability and interpretability will become vital to improve clinician and patient trust in machine-based decisions. Currently, only three graph-based AI models have explainability; however, these three methods are difficult to interpret without technical knowledge.

**WHAT THIS STUDY ADDS**

- This study describes the development and comparison of four methods to explain the predictions from a graph-based hip replacement risk prediction model visually.

**HOW THIS STUDY MIGHT AFFECT RESEARCH, PRACTICE OR POLICY**

- Results from this study demonstrate four post hoc explainability methods, with simple visualisation outputs to help explain internal AI decision-making, which does not require a technical background to interpret.

## Introduction

Primary hip replacement can be life-changing for people, improving both joint function and quality of life by reducing pain.^1^ Primary hip replacements have risen in the UK from 48 700 per year in 2006 to 101 828 per year in 2019, which is mostly due to an ageing population and rising rates of hip osteoarthritis.^1 2^ The increasing demand for hip replacements is resource intensive within the UK’s National Health Service (NHS), leading to long waiting lists.^3^ If we can predict who requires a replacement well in advance, we can suggest preventative care or pain management and triage patients. In our previous work, we predicted hip and knee replacement risk 1 and 5 years in advance from electronic health records (EHRs) using temporal graph-based convolutional neural networks (TGCNNs).^4^ Compared with traditional prediction models, this type of neural network is unique in that the entire patient history (including the sequence and timing of events) is used.

The complexity of the model means that one model prediction from the TGCNN is not readily explainable. Simple approaches, such as feature importance, are not possible. The work described here focuses on adding post hoc explainability methods to the existing TGCNN model and investigating their effectiveness in a cohort of clinicians.

Contributions: (1) We use four post hoc explainability methods to visually show TGCNN prediction decision influence for individual patients. (2) We create an interactive graph visualisation that allows clinical users to interact with historical patient EHRs. (3) We evaluate these graphs using human/clinician evaluation feedback alongside edge detection bias (EDB), sensitivity and sparsity. (4) We perform subgraph analysis to find frequent subgraphs that have an impact on the model decision.

## Related work

Post hoc explainability methods include backpropagation-based,^5^ approximation-based and perturbation-based methods.^6–8^ Ante hoc methods include attention-based, causality-based and physics rule-based models. Liu *et al* categorise methods for explaining graph neural networks (GNNs): model-agnostic methods, including subgraph-based approaches, which identify influential subgraphs,^9^ feature attribution methods that assess node/edge importance and their interactions^7^ and counterfactual explanations that explore minimal changes leading to different predictions. Model-specific methods include attention-based models, graph masking approaches and self-explaining GNNs that integrate explanation generation into their predictions.

Contrastive gradient-based saliency maps generate heatmaps by differentiating the output of the model with respect to the model input, where the positive values in the heatmap represent the input feature importance.^10^ Class activation mapping (CAM) provides a slight modification for convolutional neural networks (CNNs) by taking the gradients of the output of the model with respect to the output of the final CNN layer; this is thought to return more meaningful features in the input data. However, CAM requires the final CNN layer to be just before the final output layer of the model, with only a global average pooling layer in between. Gradient-weighted CAM (Grad-CAM) improves on this further by allowing layers between the final CNN layer and the output by weighting feature maps, while also considering feature map activations rather than just gradients.^10 11^ Explainability methods, including contrastive gradient-based saliency maps, CAM, Grad-CAM, Grad-CAM Avg and excitation backpropagation, have been adapted from CNNs to graph CNNs.^10^

**Figure 1:**
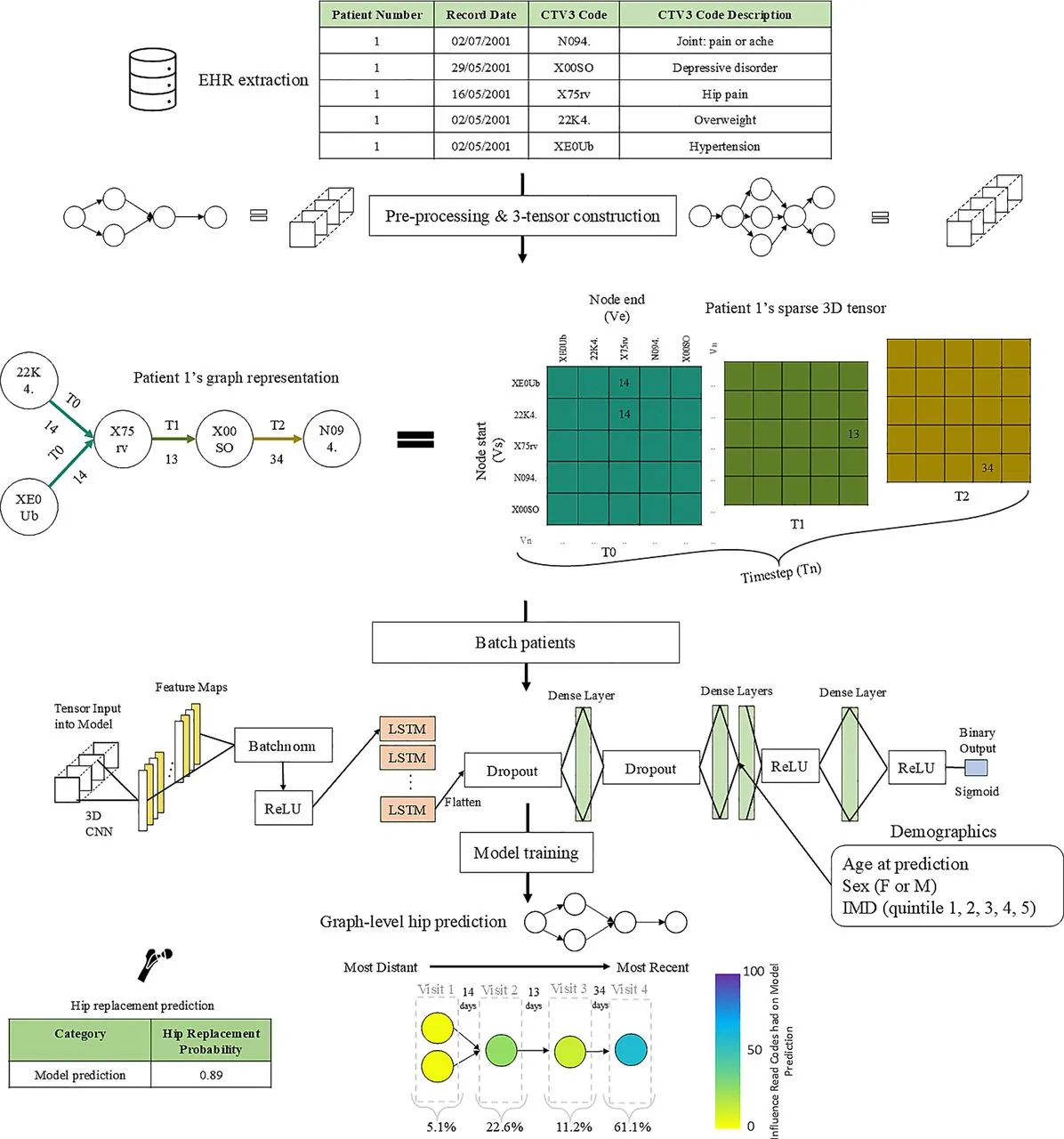
Process from a sample patient EHR to graph representation, model architecture and explainable prediction: a patient with five clinical codes over four visits is predicted with 0.89 probability to need a hip replacement within 5 years. CNN, convolutional neural network; CTV3, clinical terms version 3; IMD, index of multiple deprivation; EHR, electronic health record; ReLU, rectified linear unit; LSTM, long-short term memory.

We conducted a literature review (online supplemental material appendix A), which identified three papers that implement graphs and artificial intelligence (AI) with explainability methods using EHRs for healthcare prediction.^12–14^ A PRISMA diagram showing a flow chart of the literature search can be found in figure 1 of online supplemental appendix A. All of these papers used attention mechanisms and incorporated temporality into their explainable models.

10.1136/bmjdhai-2025-000136.supp1 Supplementary data

Su *et al* presented the GATE model, a graph-attention augmented temporal neural network for medication recommendation, which captures relationships among symptoms, medications and temporal changes in medical history at each admission. The graph attention mechanism enables the model to prioritise important aspects of the medical history, particularly the temporality. Similarly, Sun *et al* integrated medical event temporality, frequency and attention mechanisms on graph structures to predict outcomes such as mortality and readmission, with graph nodes representing diagnoses, symptoms and treatments.^13^ Smith *et al* use GNNs, recurrent neural networks and attention mechanisms to predict patient survival times.^14^

## Methods

### Model

The original TGCNN model was trained using NHS EHR data from ResearchOne, managed by The Phoenix Partnership.^15^ The ResearchOne data are not distributable under licence and are not publicly available. The code for the following methodology can be found here (https://github.com/ZoeHancox/explainable_TGCNN). This work adapts the existing TGCNN model with four explainability methods, using a training cohort of 5243 patients who have a hip replacement and 5243 who do not have a hip replacement within a 5-year window. For more details on the cohort, we refer readers to our previous work.^4^

Figure 1 illustrates the process from data to prediction to explanation and the four explainability methods we investigated are described below.

### Maximum activation difference

The TGCNN model looks at the input data and breaks it down into feature maps that highlight various patterns in the patient history. For each pattern, the model checks how strongly it reacts to patients (called ‘activation’) who either get a hip replacement or do not get a hip replacement. It calculates the average strength of this reaction for each group, then compares these averages to see which feature map shows the biggest activation difference between the two groups. The most distinctive feature map is considered the most useful for understanding what separates the groups and is used in the explainability methods to help explain the model’s decisions.

The TGCNN model creates M feature maps out from its 3D CNN layer. Let $A^{m}$ be one of the M feature maps from the 3D CNN layer, the mean activation is calculated as shown in equation 1.

(1) $${\bar{z}}_{m,c}=\frac{1}{\left|S\right|}\sum_{i\in S}max\left(z_{i}\right)$$

where $z_{i}$ is the vector of activations for the i -th patient, m is the feature map number and $S=\left\{A_{i}^{m}\;where\;y_{i}=c\right\}$ is the set of all feature maps where the collection of feature maps in S is grouped by class, $A^{m}$ , for the patients in class c .

The absolute difference in mean activations between the classes for each feature map is:

(2) $$\Delta A^{m}=\left|{\bar{z}}_{m,c_{1}}-{\bar{z}}_{m,c_{2}}\right|$$

where $A^{m}\in R^{T_{1}}$ is the flattened feature map for the m -th filter, $c_{1}$ is class 1 (hip replacement received) and $c_{2}$ is class 2 (no hip replacement received).

### Explainability methods

#### Grad-CAM

We adapt the Grad-CAM methodology^16^ for our 3D graph representations. We show activation along the axis k , showing which time steps were activated. Given activated time steps, we can observe which primary care visits contributed to the prediction.

We save the optimised weights from the trained TGCNN model and then load them into a Grad-CAM model. We apply the Grad-CAM model to a patient’s graph, $G_{p}\in R^{N\times N\times T_{2}}$ , where N is the number of clinical codes and $T_{2}$ is the number of visits, and retrieve the gradients to calculate the localisation map: the size $T_{1}$ of each 1D CNN feature map is:

(3) $$T_{1}=\frac{T_{2}-\text{filtersize}}{\text{stride}}+1$$

where filter size refers to how many patient visits the model examines at once using each filter and the stride indicates how far the filter shifts forwards (in visits) each time it performs a calculation.

Grad-CAM computes the gradient of the output, Y , with respect to the feature map $\nabla_{A^{m}}Y:\left\langle \frac{\partial Y}{\partial A_{1}^{m}},\ldots ,\frac{\partial Y}{\partial A_{T_{1}}^{m}}\right\rangle$ , where $A_{e}^{m}$ denotes the e -th element of the feature map ( $e:0§amp;lt;e\leq T_{1}$ ) and $\nabla_{A^{m}}$ is the vector of partial derivatives with respect to all the elements in the feature map m .

It then averages the gradients over each single feature map to get the weight $\alpha_{m}$ : 

(4) $$\alpha_{m}=\frac{1}{M}\sum_{e}\frac{\partial Y}{\partial A_{e}^{m}}$$

where $\alpha_{m}$ is the weight for the m -th filter.

In standard Grad-CAM, the weighted sum of the feature maps using the weights $\alpha_{m}$ are calculated and passed through a ReLU. This ignores negative weights, which are associated with decreases in risk. Alongside this, we use the following modified version (method (b) Grad-CAM (abs)) to observe the difference between the methods:

(5) $$L_{\text{Grad-CAM}}=\text{abs}\left(\sum_{m}\alpha_{m}A^{m}\right)$$

The resulting 1D localisation map $L_{\text{Grad-CAM}}$ can be used to understand the input sequence location most important for the classification decision.

An average weight for each time step is calculated using the time steps that correspond to the filter window. To assign the $L_{\text{Grad-CAM}}$ values to the original patient graph $G_{p}$ , we spread the value equally across the *d* time steps in $G_{p}$ which form each element of V . Where $V\in R^{T_{2}}$ is the vector of $L_{\text{Grad-CAM}}$ values at each time step (visit) k .

Let $w\in R^{T_{2}}$ be the weight at each time step in $G_{p}$ .

Let $X_{m}$ be the indicator function and $v_{m}$ is each value within V .

$$X_{m}(i)=\left\{ \begin{array}{ll} 1\wedge , & \text{if }k_{i}\in G_{p}\text{ contributes to }v_{m},\\ 0\wedge , & otherwise. \end{array}\right.$$

then

(6) $$w_{i}=\frac{1}{d}\sum_{m=1}^{T_{1}}X_{m}\left(i\right)v_{m}$$

#### Fm-act graphs

The feature maps from the CNN layer can indicate the patterns learnt by the TGCNN model. We propose the fm-act methodology as below:

Extract the feature maps $\left(A_{m}\right)$ from the 3D CNN layer of the TGCNN model.

Let A denote one of the following summaries: the flattened feature map with the strongest class differentiation $\max(\Delta A_{m}),\;mean(A),\;or\;median(A)$ .

Map the feature map weights to the time steps and get an average of the weights for each sliding window recurrence on each time step:

(8) $${\bar{W}}_{k}=\frac{1}{\left|W_{k}\right|}\sum_{\left(i\right)\in W_{k}}A_{i}$$

where $W_{k}$ is the set of all sliding windows that include time step k .

Normalise the weights to get the percentage influence of each time step: $W_{\text{\%}}^{k}=\frac{{\bar{W}}_{k}}{\sum_{k}W_{k}}\times 100$ .

#### Edge-act graphs

As another way of obtaining feature importance, we can look at the filters from the CNN layer and use these alongside the original input graph to find edge importance.

Extract the filters: $F=f_{1},f_{2},\ldots ,f_{m}$ from the 3D CNN layer of the TGCNN model.

Let f denote as one of the following three options: the filter $f_{m}$ with the feature map with the strongest class differentiation the feature map with the strongest class differentiation ( $max(\Delta A_{m}))$ , the mean of the filters $\left(\text{mean}\left(F\right)\right)$ or the median of the filters $\left(\text{median}\left(F\right)\right)$ . Compute the edge-act via a 3D sliding window:

(9) $$E_{i,j}=\frac{1}{\left|Wi,j,k\right|}\sum_{\left(a,b,c\right)\in Wi,j,k}\left(G_{a,b,c}⊙f_{a,b,c}\right)$$

where $W_{i,j,k}$ is the 3D sliding window over the input graph G for each filter position $\left(i,j,k\right)$ and ⊙ is elementwise multiplication.

Normalise the weights to get percentage influence of each edge: $E_{\%}^{i,j}=\frac{E_{i,j}}{\sum_{i}\sum_{j}E_{i,j}}\times 100$ .

### Interactive visualisations

We use Plotly (V.5.23.0) and NetworkX (V.3.2.1) to plot interactive graphs. A patient’s individual graph is shown where the nodes are the clinical codes, stacked clinical codes occurred during the same visit and the edges show the days between visits. A patient’s risk of requiring a hip replacement in 5 years is also provided.

### Graph visualisations metrics

Saliency maps may not always be reliable for understanding model decision-making as they rely on intuition, have poor falsifiability^11^ and tend to represent noise rather than signals.^9^ For this reason, we score the methods using the evaluation metrics below.

#### Sensitivity

For the Grad-CAM and fm-act methods, we add a node with a random clinical code assignment to a random existing visit 10 times, then obtain the mean L1 distance between the original and edited visit. For the edge-act model, we randomly change a node’s clinical code and compare the influence of the connected edge going into the node to the original edge influence. Higher values determine higher methodology sensitivity.

#### Edge detection bias (EDB)

We changed the model weights by randomly adding noise (with the same mean and SD as observed from the trained model weights) to observe changes in the heatmaps, which are used to suggest the observed influence of edges and visits. If EDB (false saliency) is present, then the heatmaps will not change or will be very similar. Ideally, the percentage influence will be dependent on the weights of the model. We calculate the mean absolute error (MAE) for the difference between the heatmap from the trained model and the heatmap from the random weighted model for each of the methods, then get the mean and SD of the differences across the patients. A higher EDB value indicates false saliency is less likely.

#### Sparsity

We calculate node and edge weight sparsity by binarising the heatmaps, setting values over 0 to 1 and others to 0. The sparsity for each graph is defined as the percentage of non-zero entries. A higher sparsity value reflects a greater number of nodes or edges that contribute nothing (ie, have zero weights) to the model’s prediction,^10^ which may be better for larger graphs, to make visual focus more obvious due to node/edges with values of zero not being visualised.

### Graph visualisation human evaluations

**Figure 2:**
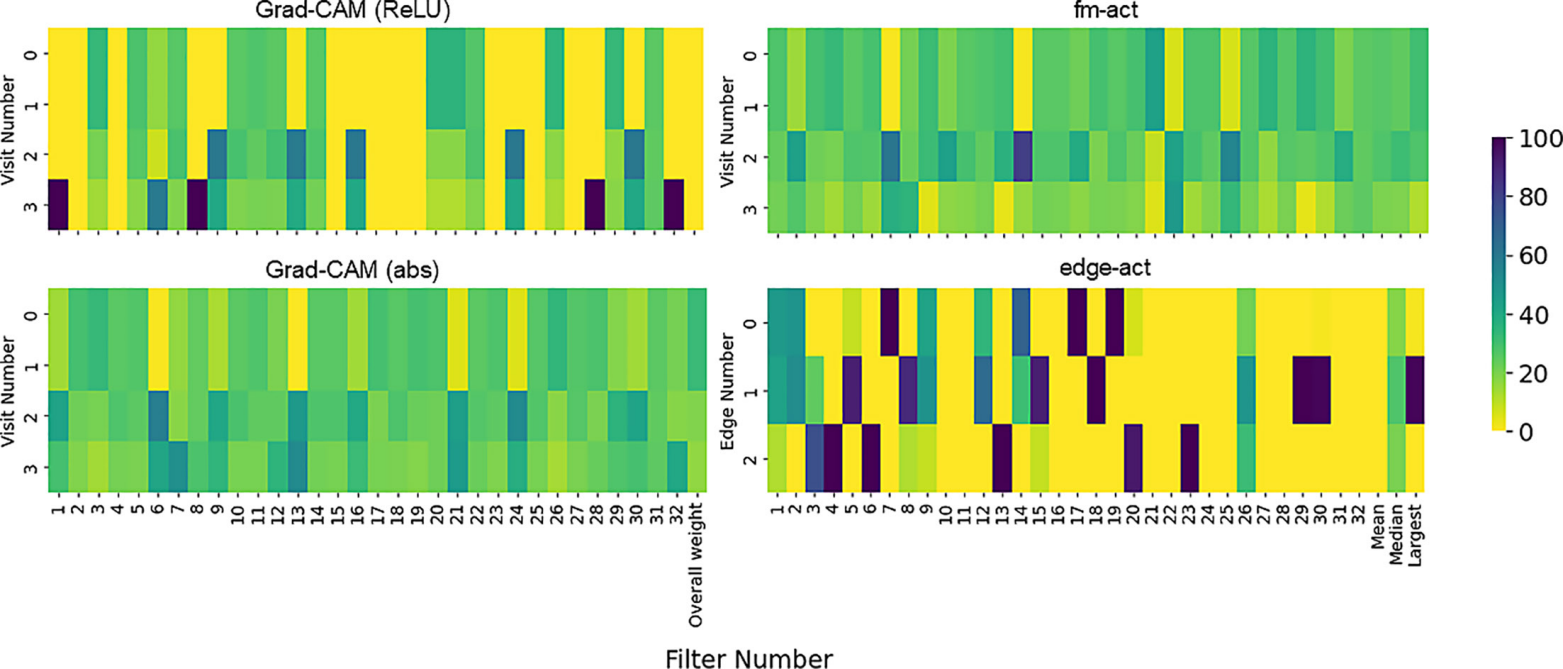
Heatmaps showing the percentage influence of features on model predictions using different methods. Grad-CAM, gradient-weighted class activation mapping; ReLU, rectified linear unit.

**Figure 3:**
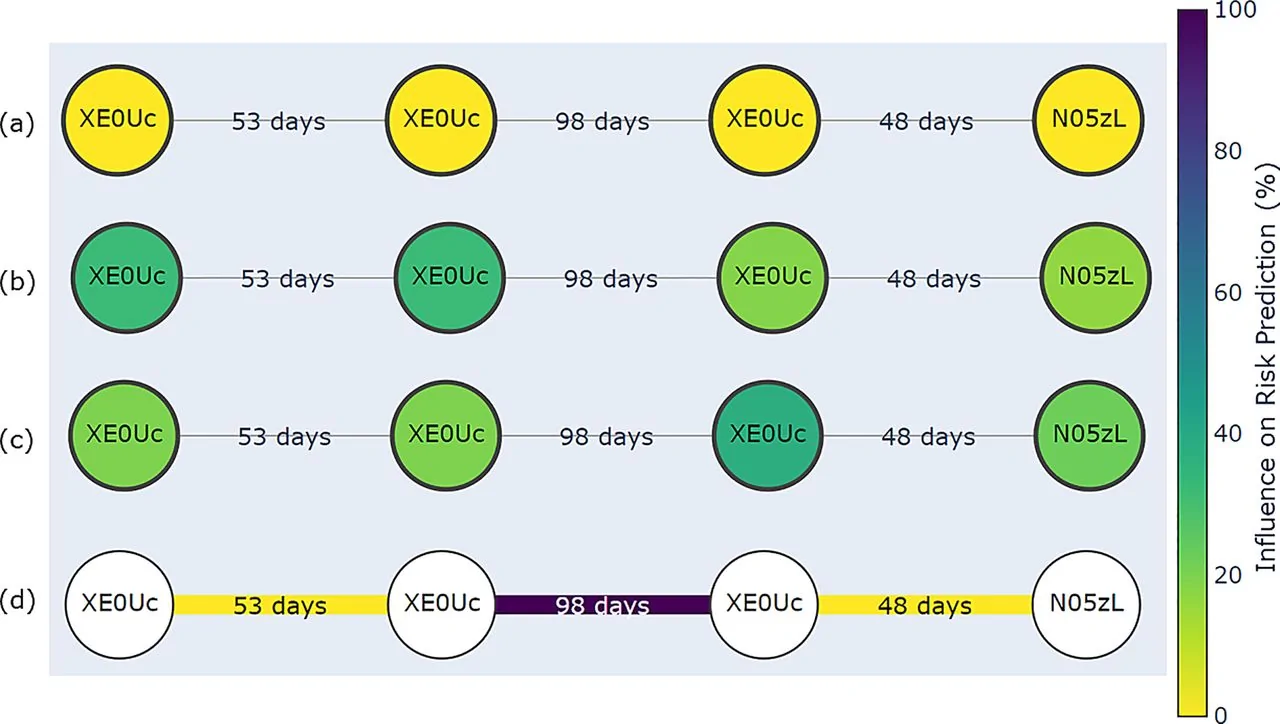
Percentage influence on features using four methods: (a) gradient-weighted class activation mapping (Grad-CAM) (rectified linear unit), (b) Grad-CAM (abs), (c) max fm-act and (d) median edge-act. Here, the patient’s predicted risk was 3.61% and they did not receive a hip replacement. Clinical code descriptions: XE0U=essential hypertension and N05zL=osteoarthritis of knee.

We carried out qualitative human evaluation studies to assess the interpretability versus truth trade-off of the four explainability methods, while gauging user interaction experience.^17^ We asked seven clinicians to complete a survey which showed the four graph methods visualised (a=Grad-CAM original, b=Grad-CAM (abs), c=fm-act and d=edge-act) for five different patient cases. A clinical vignette was provided to explain the visualisation (figures 2 and 3) in online supplemental appendix B. The survey questions we asked the clinicians are in online supplemental appendix B; this included free-text questions and Likert scales (no statistical significance testing was carried out).

### Subgraph frequency analysis

Once we have collected the sensitivity, EDB and sparsity of each method, we select the optimum method and perform subgraph analysis on it. To analyse the frequency of subgraphs across the patients, we first find all of the edges with a percentage influence of more than 0 (we denote these as ‘activated’ nodes). We then take the collections of connected activated nodes and their connection edges, repeating this for all patient graphs. The subgraph frequency is counted by prevalence per class $N_{{+}^{s},N{-}^{s}}$ for subgraph s , then we obtain the ratio to give subgraph prevalence by class: $R_{{+}^{s}}=\frac{N_{{+}^{s}}}{N_{{+}^{s}}+N_{{-}^{s}}}$ , $R_{{-}^{s}}=\frac{N_{{-}^{s}}}{N_{{+}^{s}}+N_{{-}^{s}}}$ using equations from.^10^ Please note that the subgraphs obtained have not been established as definite patient trajectories that influence hip replacement risk; we use these subgraphs to illustrate how the model infers its prediction globally.

## Results

### Methodology comparison

The distribution of maximum activation values from each CNN feature map for all patients exhibits non-Gaussian characteristics with noticeable skewness. Among the feature maps, feature map 30 demonstrates the most significant difference in activation between the classes.

Table 1 shows the results from evaluating the different methodologies, with the average scores being calculated across 10 486 patient graphs.

**Table 1:** Evaluation results mean (SD)

		Sensitivity ↑	EDB MAE ↑	Sparsity ↑
(a) Grad-CAM	ReLU	4.59 (5.94)	4.40 (10.92)	0.30 (0.43)
(b) Grad-CAM	Abs	5.80 (5.90)	2.16 (4.53)	0.00 (0.00)
(c) Fm-act	Mean	6.05 (6.19)	2.25 (1.85)	0.00 (0.00)
	Median	5.96 (5.75)	1.65 (1.32)	0.00 (0.00)
	Max	6.18 (5.95)	0.03 (0.03)	0.00 (0.00)
(d) Edge-act	Mean	25.00 (23.97)	9.82 (21.07)	0.53 (0.32)
	Median	23.64 (23.26)	4.40 (4.56)	**0.55** (0.31)
	Max	23.78 (23.20)	**15.89** (26.77)	0.51 (0.33)

Bold values indicate the best performing method for each of the key performance metrics.

EDB, edge detection bias; Grad-CAM, gradient-weighted class activation mapping; MAE, mean absolute error; ReLU, rectified linear unit.

Figure 2 shows the heatmaps for one patient with the four explainable methodologies, indicating the influence of each visit or edge depending on the filter and method.

### Interactive visualisations

Figure 3 shows the graphs used to show an example patient pathway and influence healthcare records have on model prediction. When these models are interacted with as HTML files, users can hover over nodes (Grad-CAM and fm-act graphs) and edges (edge-act graphs) to show the percentage influence on the model decision and the clinical code descriptions. Interactive versions of these graphs (https://zoehancox.github.io/graph-survey/index.html) are available for readers to explore.

### Clinical feedback

Some patients had long EHR histories, which led to complex graph visuals, while others were simpler and easier to visualise without zooming or panning to specific areas of the graph.

Overall, the fm-act method (c) was voted as the easiest graph to interpret (n=15/35) and Grad-CAM (abs) (b) the hardest (n=1/35) (online supplemental appendix C—figure 6). There were varying opinions on the effectiveness of methods in highlighting key factors influencing model predictions across different patient graphs, with the two longest patient graphs having the worst feedback (online supplemental appendix C—figure 4). Overall, satisfaction with the methods decreased as the length of the patient history increased (online supplemental appendix C—figures 4 and 5). Patient graphs 2 and 3 had the most agreement from clinicians for expected trajectory alignment. There was 57% agreement in all the patient graphs that trajectories met expectations; however, some methods were selected as not aligning with expectations (online supplemental appendix C—figure 5). When we asked clinicians to what extent the graphs supported understanding the model’s decision-making process, one said it ‘greatly supported’, two said it ‘moderately supported’, three said it ‘slightly supported’ and one scored it as ‘neutral’. Three clinicians felt that these graph visualisations had ‘neutral’ input for aiding decision-making in a clinical setting, one thought they were ‘very useful’, one thought they were ‘somewhat useful’ and two clinicians felt they were ‘somewhat useless’. These graphs appeared useful to demonstrate model decision-making but were less helpful for aiding clinical decision-making.

The survey feedback highlighted that the fm-act method (c) stands out for its subtle differences and ease of engagement, with better colour discrimination between nodes. Methods a–c (Grad-CAM (ReLU), Grad-CAM (abs) and fm-act) more effectively addressed model complexity. However, there was a general consensus that the colour scale should be given more emphasis across all methods. Method d (edge-act) received mixed reviews: while one clinician found it visually unappealing, another preferred it over the Grad-CAM methods (a and b) for its visual clarity. Additionally, there was some confusion from a clinician regarding the model’s decision process, specifically questioning the connection between hypertension and hip replacement.

The survey results suggest that the current graph layout is too crowded and detailed for general practitioners to effectively use within the time constraints of a primary care appointment. A visual summary of the most influential factors is recommended to facilitate quicker decision-making and patient communication. While detailed graphs are valuable for understanding model decision-making, a focus on summary risk prediction scores is deemed more practical. Simplifying the colour coding could improve clarity and distinction where percentages are similar, though this may limit the ability to compare multiple patients visually.

### Subgraph frequency analysis

**Figure 4:**
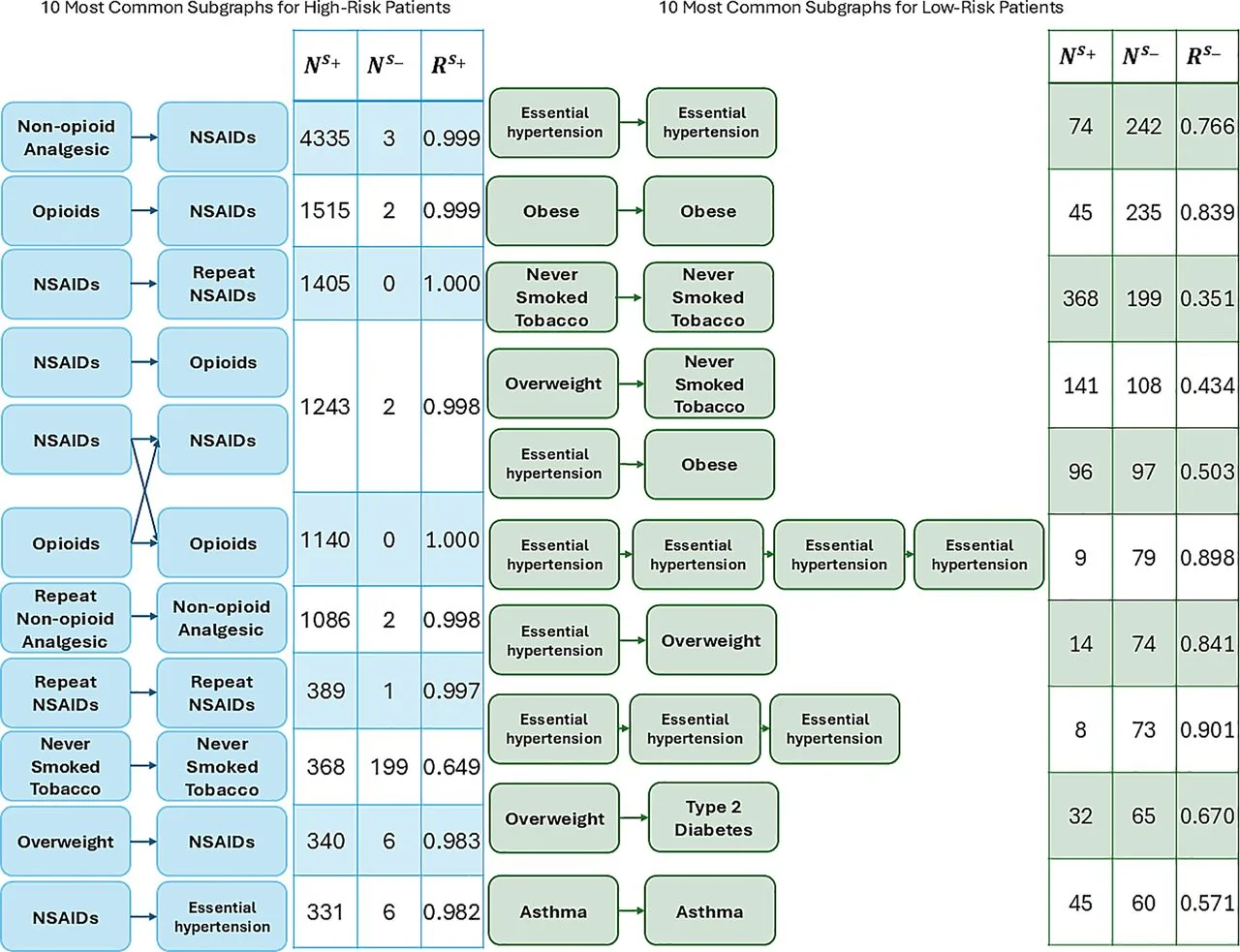
The 10 most frequent subgraphs that influence model prediction for each class. NSAIDs, non-steroidal anti-inflammatory drugs.

We collected subgraphs using the median edge-act method. A total of 13 383 subgraphs were identified. 10 594 subgraphs only appeared once (79.2%). The most frequent subgraph for patients with high hip replacement risk was Non-opioid analgesic → non-steroidal anti-inflammatory drugs (NSAIDs) prescription, with many of the most frequent subgraphs containing prescriptions. See figure 4 for the 10 most frequent subgraphs influencing model prediction for each class.

## Discussion

The four explainability methods described in this paper aim to produce a clinician-interpretable justification for each output. These methods may increase decision confidence if the results match clinical expectations. However, the absence of a plausible explanation does not imply an inaccurate model. As these methods are post hoc, important features may be spuriously correlated with replacement risk or non-causal.^18^

Explainable TGCNN models are valuable for offering intuitive insights into how clinical code pairs or GP visits affect predictions, using simple percentages. This accessibility helps medical professionals understand and trust the model. Often, ‘explainable’ methods require advanced technical knowledge, making them inaccessible to users without a machine learning or data science background.^8^

The max edge-act method gave the highest MAE between the trained/original model and the random weighted model. The fm-act method is more prone to EDB than the other methods, as shown by its smaller MAE values, and max edge-act is the least likely to have EDB.

There was a non-Gaussian distribution when comparing maximum activation differences; therefore, we discard the mean edge-act method. The fm-act and Grad-CAM (abs) methods lead to graphs with zero sparsity (table 1). This means that these graphs could be difficult to interpret if a patient has a long EHR history. The median edge-act method gave the best sparsity results, while the max edge-act method gives the best EDB and slightly higher sensitivity results.

From the results in table 1, we determined that the median edge-act and Grad-CAM (ReLU) methods provide the best visual explainability for the TGCNN model. The Grad-CAM (ReLU) model is useful for showing the influence of visits, while the edge-act model shows the influence of edges. We did subgraph frequency analysis on the median edge-act method, as its high sparsity suggests the subgraphs should be smaller and more common among individuals. The fm-act method had the most votes for clinical interpretability; however, due to a lack of sparsity, we believe this method would not be scalable for long EHRs. Clinicians favoured the graphs where the nodes were colour-coded rather than the edges; therefore, there might be future scope to adapt the edge influence onto the node colouring.

Our methodologies have the following limitations: (1) Due to the nature of these methods, they are not falsifiable without human interpretation. We cannot know if the model is predicting based on patterns that are reasonable/align with a clinician’s thought process, without clinical assessment. (2) Our models do not consider causality; however, the model may help us identify features that influence hip replacement risk that may be currently unknown to clinicians. (3) Our method compares heatmaps from an original model and a noise-perturbed version to measure EDB. However, this approach can be sensitive to the type and amount of noise used and may reflect model instability rather than the reliability of its explanations. (4) While other path-based explainable AI methods such as integrated gradients,^19^ expected gradients^20^ and manifold integrated gradients^21^ could be considered to satisfy more axioms in explainable AI, we focused on structural patterns rather than feature-level attribution. Our approaches do not aim to trace prediction paths or assign importance to individual features across patients, but rather to identify and interpret frequent subgraphs within clinical data. As such, applying path-based methods directly would require significant adaptation and did not align with our analytical goals. Future work could explore how these attribution techniques could be extended to graph-based settings for complementary insights.

Future work could involve attention mechanisms, allowing the model to focus on specific inputs during the training process. However, these methods may be significantly more computationally expensive. Where Grad-CAM focuses on class-specific influence, it is limited by its inability to provide an understanding of global patterns or relationships within the input. The feedback on our suggested methods directs our focus to scalability and dimensionality reduction in future iterations of these methodologies. Specifically, we aim to adjust how the graph visualisations are presented to clinicians, prioritising the most informative regions of the EHR history visually first.

Clinicians can use this tool to assess the 5-year risk of a patient needing a hip replacement based on their existing EHR data. For deeper insights into specific model decisions, clinicians can interact with the visualisation tools described in this paper to explore a patient’s clinical code history and identify key factors influencing predictions. This can aid in patient care decisions, such as painkiller prescribing, physiotherapy and exercise recommendations. Additionally, these methods can assist in resource planning by generating lists of patients anticipated to require surgery in 5 years. Clinicians can show these graphs to their patients, demonstrating model decision-making while providing motivation for patients to adhere to treatment plans.

## Conclusion

We use four methodologies on a temporal graph-based CNN model to improve the explainability of hip replacement risk prediction. Our edge-act method provided the best results in terms of graph sparsity, sensitivity and reduced EDB. Based on our subgraph frequency analysis, prescriptions are highly influential to model prediction. Clinicians found our visualisation techniques useful to explain model outputs.

## Data Availability

Data may be obtained from a third party and are not publicly available. The ResearchOne data used in this research are not distributable under licence and are not publicly available.
