# Supplementary material for: Explainable temporal graph-based CNNs for predicting hip replacement risk using EHR data
Source: BMJ Digit Health Ai. 2026 Jan 27;2(1):e000136. doi: 10.1136/bmjdhai-2025-000136 (PMC13528391; doi:10.1136/bmjdhai-2025-000136)
Supplement: Supplementary file 1 [file bmjdhai-2-1-bmjdhai-2025-000136-s001.pdf]

SUPPLEMENTARY MATERIALS

A LITERATURE REVIEW

A.1 Method

When reviewing the current literature in the area we asked the following research question: How is explainable AI being used, with graph models on EHRs, to help clinicians understand what features models use to predict health outcome risk?

We searched Google Scholar for articles using explainable graphs for health outcome prediction using the following search string:

(“graph neural networks” OR GNN OR “graph models”) AND (“explainability” OR “interpretability” OR “explainable AI” OR “XAI”) AND (“Grad-CAM” OR “class activation map” OR “activation mapping” OR “saliency map” OR “visuali$ation”) AND (“EHR” OR “electronic health record*” OR “medical record”).

We screened the titles and abstracts first, then full texts looking for papers that met our research question criteria. We included papers if they were written in English, published after 2010 and used graph-based models to predict health outcomes using EHRs. Grey literature and literature reviews are included in our search to get a wide overview of current methodologies being explored. We extracted relevant papers from reviews, using the snowballing technique. We provide a summary of the current existing literature in this area.

A.2 Results

Inputting the search string into Google Scholar resulted in 111 papers being returned. After title screening 56 papers were left. After abstract screening 18 papers remained. Fifteen of these 18 papers were literature reviews, from which we performing snowballing of references finding another 23 papers, totalling 26 full papers to screen. See Figure 1 for our PRISMA flowchart [20]. In total 3 papers used graphs with explainability methods using EHRs for healthcare prediction [12–14].

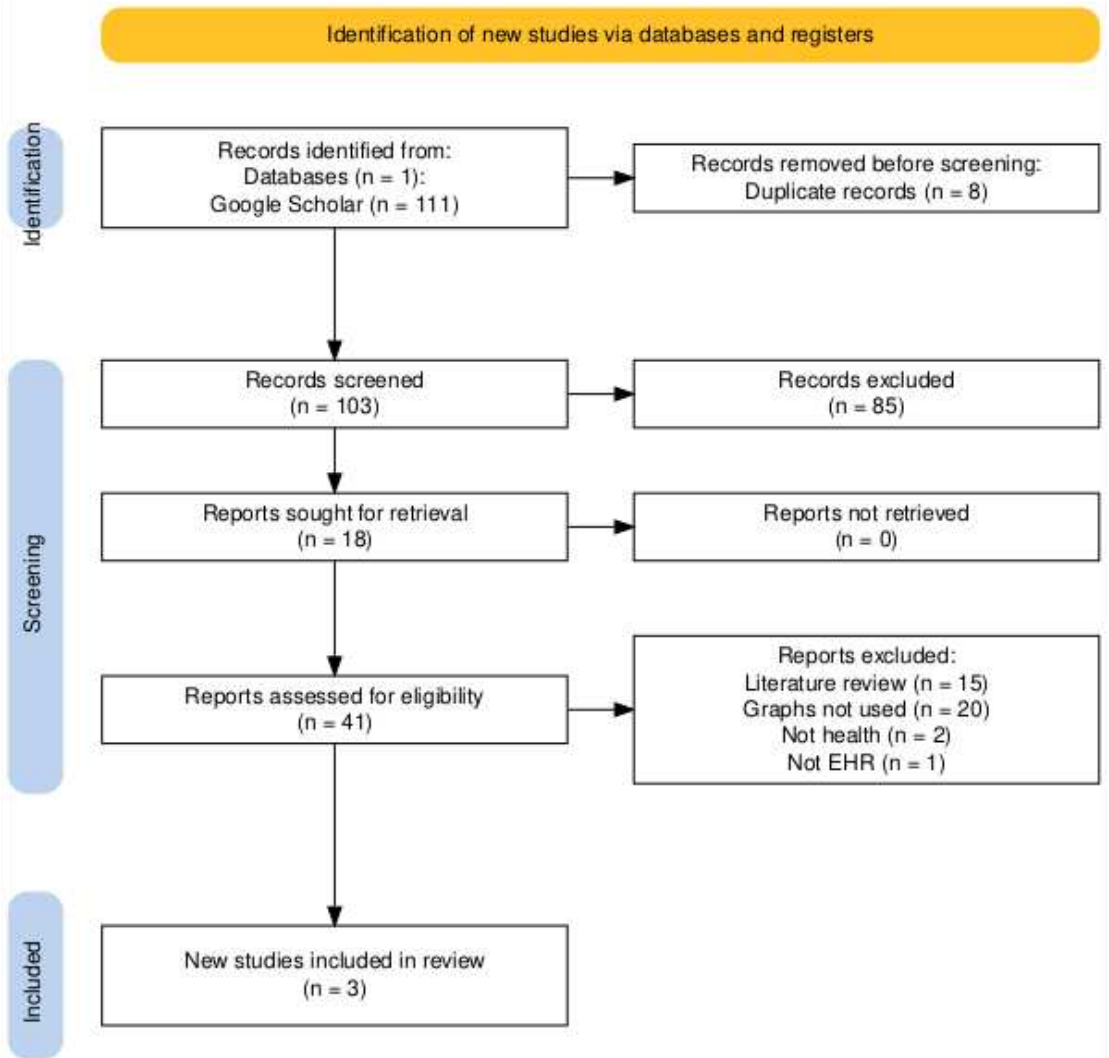

Figure 1. PRISMA flowchart of search for explainable graph methods using EHRs.

B CLINICAL VIGNETTE

We gave clinicians the clinical example as given in Figures 2 and 3.

For each patient's graph set we asked: 1) Which of these visualisations is the easiest to interpret? 2) Do any of these visualisations not align with a trajectory you'd expect? 3) Does each graphing method effectively highlight the key factors influencing the model's prediction? 4) Any additional comments about the graph?

Then we asked questions about the graphs overall: 1) How useful are these graphing methods for aiding decision-making in a clinical-setting? 2) To what extent do the graphs support your understanding of the AI model's decision-making process? 3) Which of the four graphs do you prefer for visualising AI model predictions, and why? 4) Are there any additional features or information you would like to see included in these graphs?

Clinical Vignette – Example of How to Interpret Explainable Graphs

Scenario: a patient has presented themselves for a GP appointment and is having issues with hip pain and osteoarthritis. You think this patient might be at risk of needing a hip replacement in the coming years, but you would like to quantify that risk with more certainty using explainable artificial intelligence (AI) techniques.

Their clinical record is as follows:

Date	Findings
01/08/2024	Hip pain
04/08/2024	Hypertension
08/08/2024	Hip pain<br>Hypertension
10/08/2024	Hip pain
15/08/2024	Osteoarthritis of the hip<br>Hypertension

We evaluate four different methods for visualising the features of Electronic Health Record (EHR) data that contribute to the hip replacement risk score generated by the AI model. Each method employs an interactive plot that enables users to navigate through a patient's history and identify the features (such as GP visits or Read Code pairs) that have the greatest impact on the model's predictions.

Method **a)** illustrates the influence of recordings from primary care visits on the model's prediction, focusing only on features that increase the risk score.

Methods **b)** and **c)** display the influence of these recordings on the model's prediction by considering both features that either increase or decrease the risk score.

Visualisation and labelling of methods **a**-**c**:

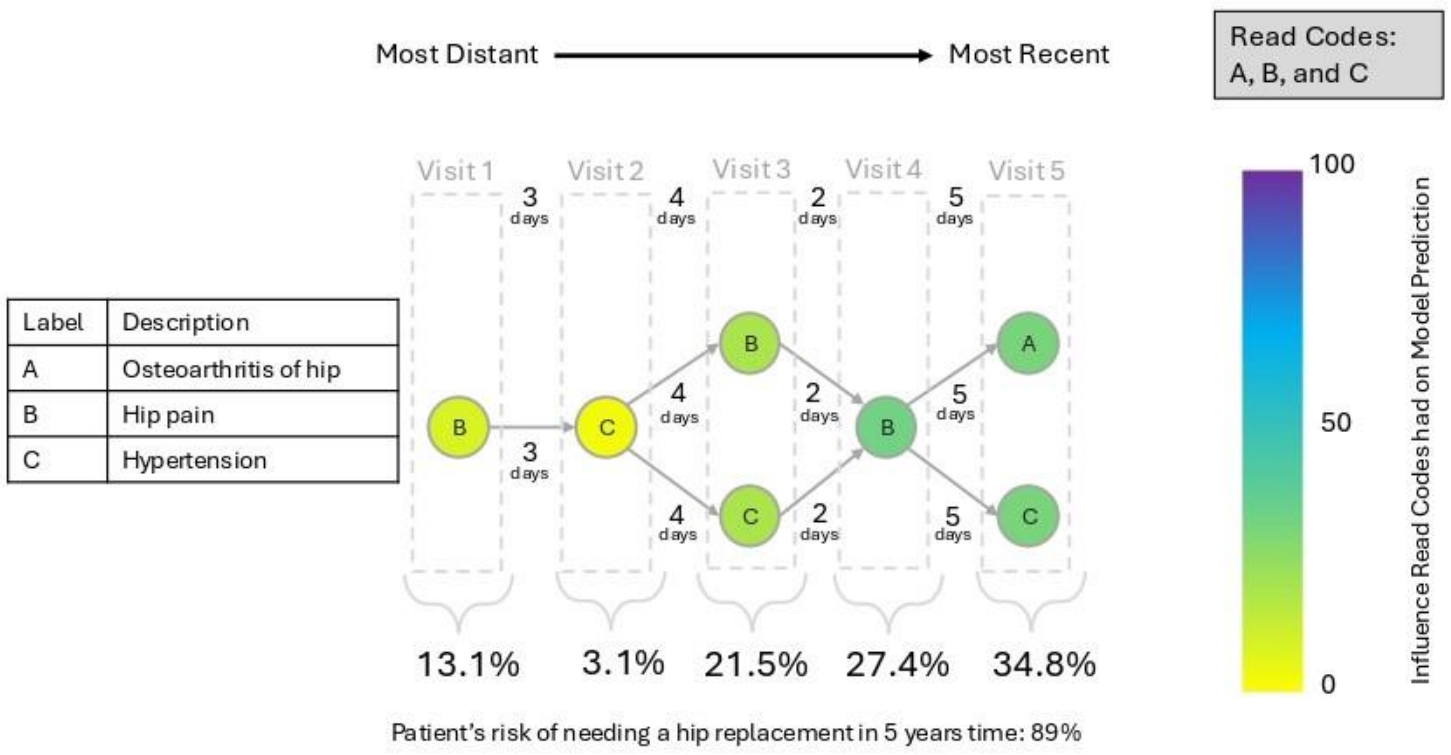

Explanation of methods **a-c** visualisation:

Visits 4 and 5 (the most recent GP visits) had the greatest influence on the risk prediction score (indicated by darker colours), with the recording of 'osteoarthritis of hip' having the most significant impact on the outcome. The patient has an 89% risk of needing a hip replacement

Figure 2. Clinical vignette page 1.

within 5 years, and the model determined the extent to which each of these visits influenced the prediction.

Method **d)** illustrates the impact of pairs of Read Codes from the individual's EHR on the prediction outcome.

Visualisation of method **d**:

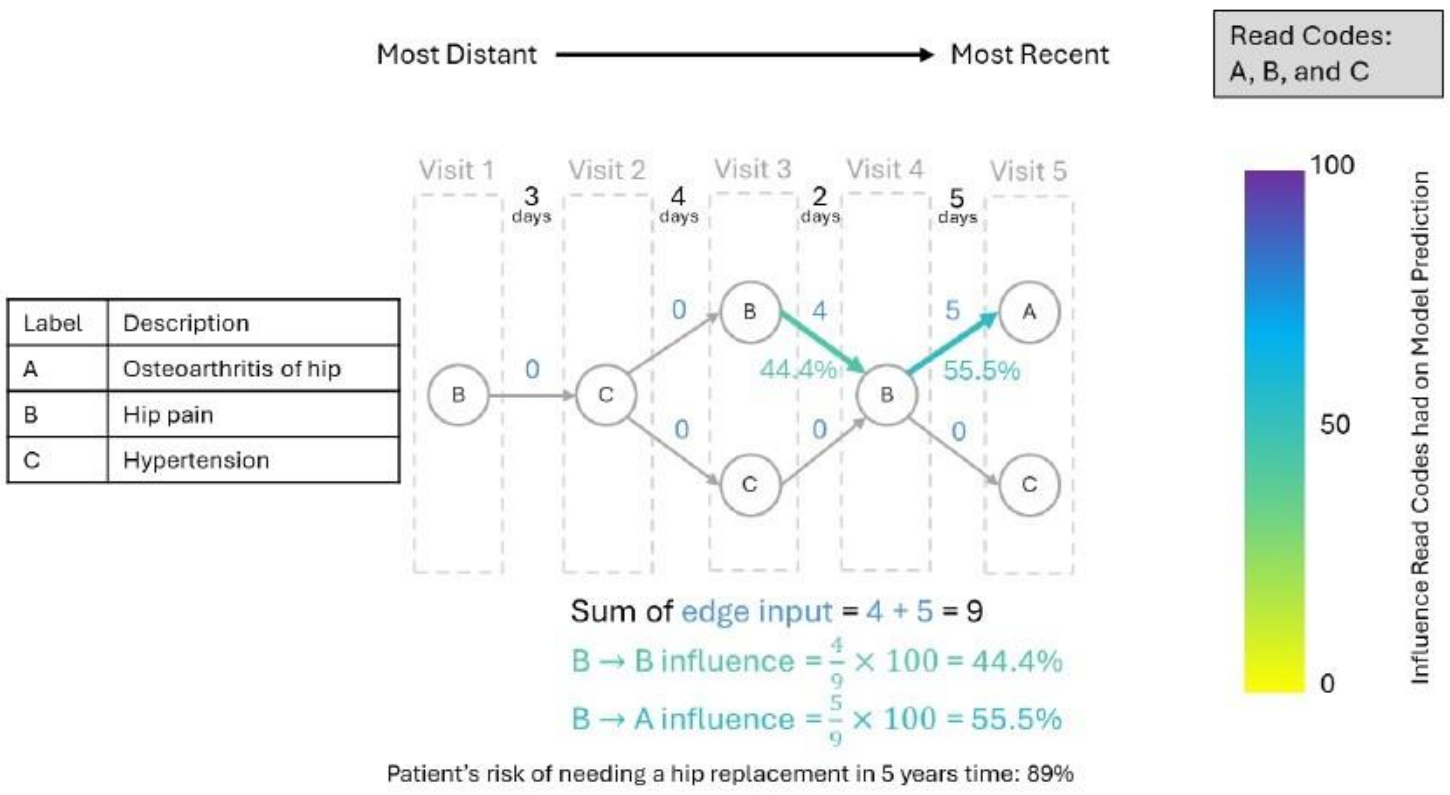

Explanation of method **d** visualisation:

The progression from a 'hip pain' recording in visit 4 to 'hip pain' in visit 5 accounted for 44.4% of the influence on the hip replacement risk. In contrast, the sequence of 'hip pain' in visit 4 followed by 'osteoarthritis of hip' in visit 5 contributed 55.5% to the hip replacement risk. The remaining interactions between Read Codes did not affect the risk score. With an 89% risk of needing a hip replacement within 5 years, the model determined that these two pairs of Read Codes influenced the risk prediction in the proportions stated.

Figure 3. Clinical vignette page 2.

C CLINICIAN FEEDBACK

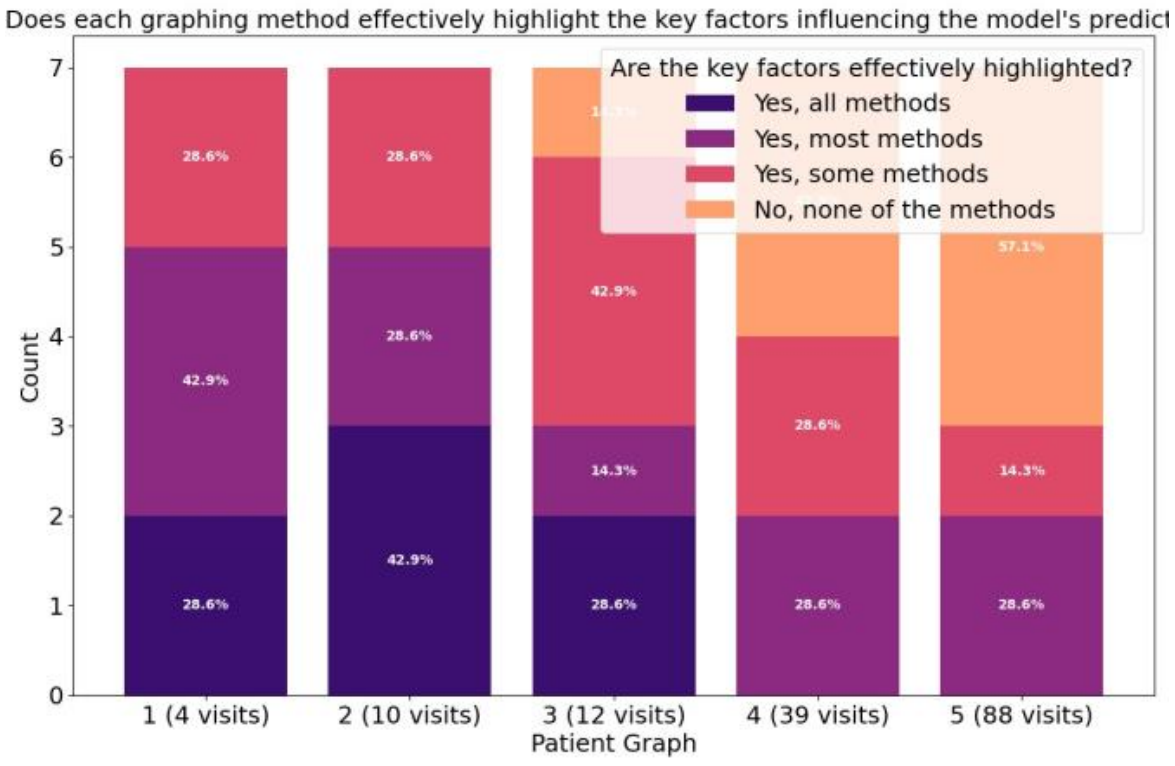

Figure 4. Clinical opinion on whether key factor influence is highlighted.

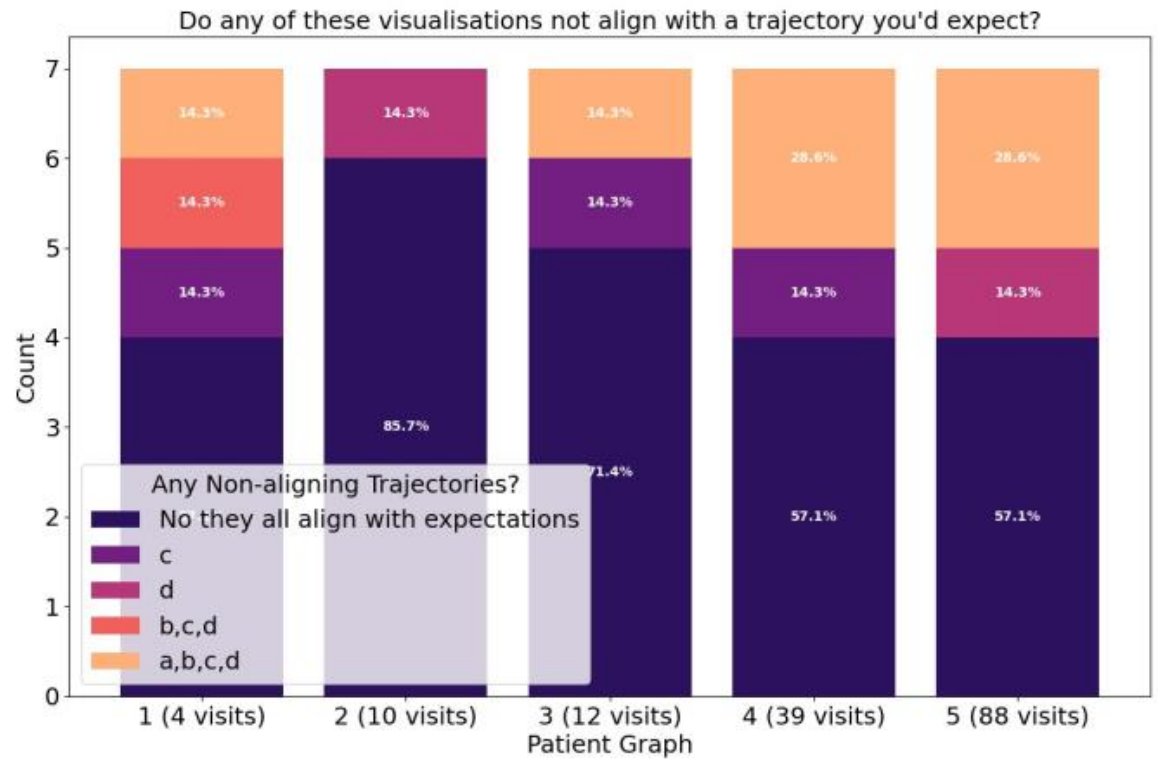

Figure 5. Clinical opinion on trajectory alignment.

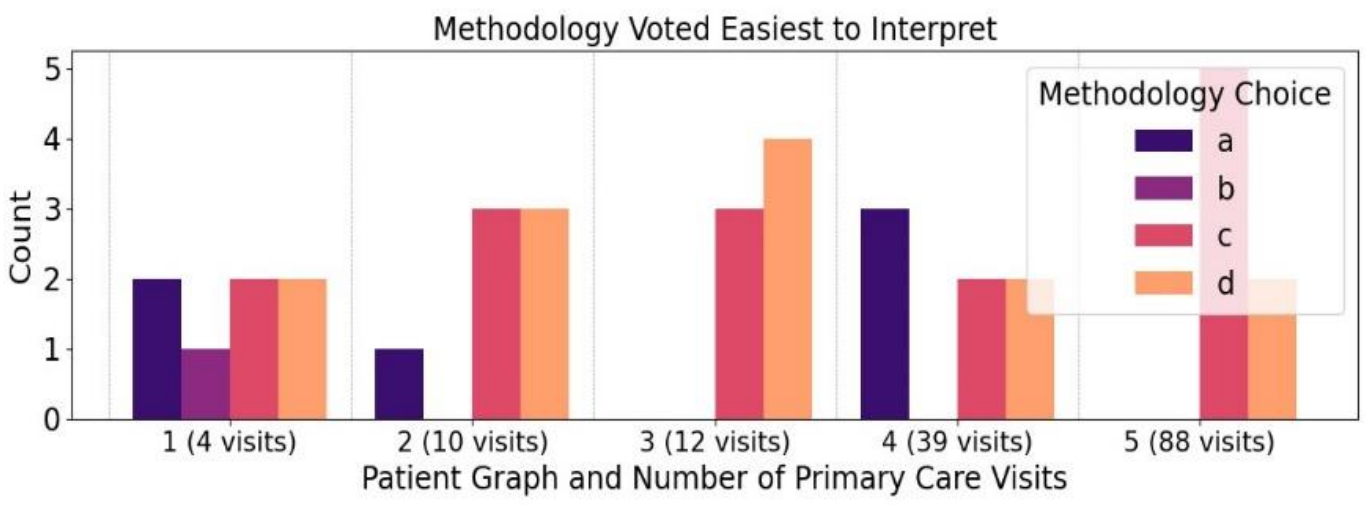

Figure 6. Which method was determined the easiest to visually interpret.
